# Benatar and Metz on Cosmic Meaning and Anti-natalism

**DOI:** 10.1007/s10790-023-09940-x

**Published:** 2023-04-19

**Authors:** Kirk Lougheed

**Affiliations:** 1 LCC International University, Klaipėda, Lithuania; 2 University of Pretoria, Pretoria, South Africa

## Abstract

David Benatar argues that one important consideration in favour of anti-natalism is based on the fact that all humans lack cosmic meaning; we will never transcend space and time such that we will have an impact on the entire universe, forever. Instead of denying Benatar’s claim that we lack cosmic meaning, Thaddeus Metz recently argues that our lack of cosmic meaning is not that significant because we ought not to regret lacking a good that we could not have in the first place. He explains the principle behind this idea in modal terms: “the closer the world in which one could access a benefit, the more reasonable are attitudes such as sadness, disappointment, regret when does not acquire it.” I argue that this principle faces a serious counterexample in the form of death. The possible worlds in which one doesn’t die are incredibly distant. Yet, it is appropriate to express deep sadness, disappointment, and regret at the fact that one must inevitably face death. Metz is wrong that we shouldn’t regret lacking a good unavailable to us in the first place. His criticism of Benatar therefore fails. While it might be objected that immortality is not good, my basic point still stands when considering the fact that our lives are not significantly longer. Benatar’s claims about the significance of our lack of cosmic meaning might not be true, but not for the reasons suggested by Metz.

## Introduction

Anti-natalism is the highly controversial view that says it is always (or almost always) impermissible to procreate.^1^ A leading proponent of anti-natalism in David Benatar connects the debate about anti-natalism to considerations about meaning. He argues that one important consideration in favour of anti-natalism is based on something all humans lack. Namely, we lack cosmic meaning; we will never transcend space and time such that we will have an impact on the entire universe, forever. It’s wrong to bring persons into existence knowing they will never have this type of cosmic meaning.^2^ Instead of denying Benatar’s claim that we lack cosmic meaning, Thaddeus Metz recently argues that our lack of cosmic meaning matters far less than Benatar suggests.^3^ According to Metz, one reason for this is that we ought not to regret lacking a good that we could not have in the first place. He explains this principle in modal terms: “the closer the world in which one could access a benefit, the more reasonable are emotions such as sadness and disappointment, when one does not have it.”^4^

After outlining the main contours of the debate between Benatar and Metz on cosmic meaning and anti-natalism, I argue that Metz’s Modal Principle faces a serious counterexample in the form of death. The possible worlds in which one doesn’t die are incredibly distant. Yet, it is appropriate to express deep sadness, disappointment, and regret at the fact that one must inevitably face death. Metz is wrong that we shouldn’t regret lacking a good unavailable to us in the first place. His Modal Principle is false and, therefore, his criticism of Benatar fails. I then explore a possible rejoinder that could be made on behalf of Metz. This rejoinder says that living forever would actually negate or detract from meaning in life because of boredom or repetitiveness. I respond by appealing to the work of J.L. Schellenberg on *deep time* to show that even if immortality is not something we should ultimately hope for, we are still right to want to live for many thousands (if not millions) of years.^5^ This is because it would take an extremely long time for boredom or repetitiveness to become a problem. Though there may be nearby worlds where some life extension is possible, the worlds where I can live for an incredibly long time are very distant from the actual world. Yet I am right to be sad and disappointed that I am not going to live for an extremely long time. This shows that Metz’s Modal Principle is false. Benatar’s claims about the significance of our lack of cosmic meaning might not be true, but not for the reasons suggested by Metz.

## Benatar on Cosmic Meaning and Anti-Natalism

David Benatar is best known for defending anti-natalism, the view that it is always (or almost always) all things considered wrong to procreate. He establishes this conclusion primarily through two arguments. The first is his *Asymmetry Argument*. According to Benatar, “there is a crucial difference between harms (such as pains) and benefits (such as pleasures) which entails that existence has no advantage over, but does have disadvantages relative to, non-existence.”^6^ The absence of pain is good even if no one experiences that good while the absence of pleasure is not bad unless someone is deprived of it. Benatar defends this asymmetry by showing that it best explains four additional procreative asymmetries, including the one that while we have a strong duty not to purposefully bring someone into existence who will suffer, we have no corresponding duty to bring someone into existence who we know will be happy.^7^ The second argument for anti-natalism is based on the idea that most lives are actually quite bad, despite the rather positive assessments of their own lives often offered by people. Benatar says that “[t]here are a number of well-known features of human psychology that can account for the favorable assessment people usually make of their own life’s quality. It is these psychological phenomena rather than the actual quality of a life that explain (the extent of) the positive assessment.”^8^ For example, the Pollyanna Principle states that people are extremely inclined towards optimism in their judgments. We tend to recall good experiences more than bad ones, which biases not only our judgments about the past but also how we envision the future.^9^ These are *philanthropic* arguments for anti-natalism because they focus on the harm done to individuals who are brought into existence. It’s worth observing, however, that Benatar has also offered a *misanthropic* argument for anti-natalism based on the harm that humans do once they are brought into existence.^10^ Such harms include those that humans do to each other, non-human animals and the environment.

Benatar’s Asymmetry Argument is by far the most discussed in the literature. For example, the distinction between a life worth starting and a life worth continuing has been questioned.^11^ Others have suggested the four additional asymmetries are actually fundamental and so not explained by anything else,^12^ that goodness and better-ness are linked conceptually which ultimately defeats the asymmetry^13^ and that there is an equivocation between personal and impersonal goodness in the argument.^14^ Christine Overall has made the general claim that Benatar’s anti-natalism could be harmful to women,^15^ with Benatar responding that his arguments very likely support the goals of feminism.^16^ Benatar’s misanthropic argument has not received nearly as much attention though recently I have made connections between it and ideas in African ethics.^17^

Other arguments for anti-natalism include the idea that it is impermissible to procreate because individuals cannot consent to the harm they will experience by being brought into existence,^18^ that procreating creates a prospective victim which is immoral,^19^ and that procreation involves exploiting a baby in order to procure a fully formed adult.^20^ Recently, ideas in anti-natalism have brought anti-natalism into conversation with ideas in artificial intelligence and futurism.^21^

Though the ideas mentioned above warrant continued attention, my focus is on a different argument for anti-natalism that has been developed by Benatar but has received significantly less attention to date. Benatar argues that another reason in favour of anti-natalism is based on the idea that all humans necessarily lack cosmic meaning. Now, the recent literature on meaning typically distinguishes between two different kinds of meaning. First, the meaning *of* life tends to denote questions about whether the human species as a whole has a meaning. Second, work on meaning *in* life asks if an individual’s life is meaningful.^22^ Much of the literature focuses on answering questions about the latter. We’ll see that though Benatar’s argument is about the lack of meaning available *in* life for individuals, his claims are meant to apply to all humans. Theories on meaning in life tend to fall into two broad categories of supernatural and naturalistic. Extreme supernatural theories of meaning say God’s existence is necessary for meaning in life, perhaps (though not necessarily) exemplified by assigning purposes for us to fulfill.^23^ More moderate versions of supernaturalism say that in one way or another God’s existence would enhance the meaning in an individual’s life.^24^ As with supernaturalism, naturalist theories of meaning come in both extreme and moderate versions. Proponents of moderate versions claim that a meaningful life is available in a naturalistic world even if God (or some other spiritual entity, or having a soul) would enhance meaning.^25^ Extreme versions of naturalism claim that God or a spiritual realm would make our lives less meaningful or render them altogether meaningless.^26^ These distinctions between extreme and moderate naturalism can be cross-divided into the two categories of subjectivism and objectivism. Subjectivists deny that there are ‘invariant standards’ of meaning that span across human minds, maintaining that what is meaningful is subject-relative (i.e., relative to an individual’s goals, desires, plans, etc.). Objectivists instead say that there are ‘invariant standards’ for meaning in life that they are (partly) mind-independent. They do not depend solely on a person’s desires or goals, etc.^27^ We’ll see that the type of meaning Benatar seems to have in mind is naturalistic and objective.

In the following, I mostly summarize Metz’s explication of Benatar’s argument since it is Metz’s criticisms of it that concerns me. Benatar writes that “once you believe this whole thing is ultimately pointless, it is ridiculous to generate more adversity-facing meaning-seekers.”^28^ While terrestrial meaning might be possible for persons, they cannot have cosmic meaning that goes beyond the world and their finite lives. Metz explains that “[a] cosmic meaning for Benatar would involve transcending the limits of space and time, so that we could make a positive difference to the entire universe and do so forever, not just a small portion of the planet we are on for about 80 years if we are lucky.”^29^

Here is Metz’s standardized interpretation of Benatar’s argument:

Benatar’s Argument for Anti-Natalism from Our Lack of Cosmic Meaning

1. The most important sort of meaning a life could have is a cosmic one.
2. No human life can in fact exhibit a cosmic meaning.
3. Lacking the most important sort of meaning is very bad for life.

Thus,

(4) There is (some) moral reason not to create a life that lacks cosmic meaning.

Therefore,

(5) Anti-natalism is (probably) true.^30^

While Metz never explicitly says it, I think he intends (1) through (4) to provide strong inductive reasons for anti-natalism. However, what I say below will apply equally to deductive versions of the argument too.

Regarding premise (1), Benatar understands “the concept of meaning in life is in terms of transcending limits.”^31^ Metz explains that:

The idea is that a life is more meaningful, as opposed to say, happy, the more it “transcends one’s own limits and significantly impacts others or serves purposes beyond oneself.” According to Benatar, many (although not all) human lives are able to transcend limits that separate them from other human beings or human goods. Many of us can develop romantic attachments, rear children with love, contribute to charity, share knowledge, and make similarly terrestrial accomplishments.^32^

For Benatar, these are sources of meaning from the human perspective, not from the perspective of the universe. In order “[t]o have meaning from the perspective of the universe would include (indeed, by definition, for Benatar) significantly impacting others throughout the cosmos or serving purposes that range over it.”^33^ Such meaning could be conferred by God or perhaps through having a positive impact on an extraterrestrial alien species. For anything one does, it’s possible to ask why it matters. In order for something to have cosmic meaning, such queries would always have to terminate in the claim that it “would transcend all limits to the point of extending throughout the cosmos in all places and in all (future) times.”^34^

With respect to premise (2), Benatar insists that no human lives have cosmic meaning, writing that “[e]arthly life is thus without significance, import, or purpose beyond our planet. It is meaningless from the cosmic perspective.”^35^ Benatar is also doubtful that it’s even *possible* for our lives to have cosmic meaning. This is because he affirms naturalism, and thus rejects the existence of God. It is also because “it appears that the universe is devoid of other physical life, and that, even if it is teeming elsewhere beyond our detection, we can never be in a position to influence it, let alone for the better and forever.”^36^

Finally, regarding premise (3), Benatar describes the lack of meaning as a cost that we should regard as unfortunate, regret that it is the case, and feel sadness over.^37^ (4) follows from (1) through (3) and (5) follows from (1) through (4).

## Metz’s Criticisms of Benatar

In this section, I outline Metz’s criticisms of Benatar on cosmic meaning. While others have tried to show that premise (2) of Benatar’s argument is false, Metz simply grants Benatar the claim that our lives do not exhibit cosmic meaning. He, therefore, grants premise (2) of Benatar’s argument, assuming both that God does not exist and that aliens do not exist (or if they do, that we cannot interact with them in the relevant ways). Instead, Metz attempts to “cast doubt on the ideas that cosmic meaning is all that important and that its absence merits negative reactive attitudes supporting the choice not to create human lives that would in every case lack such meaning.” 38 Metz, therefore, intends to object to premises (1) and (3) of Benatar’s argument.

Metz’s first objection appears to target premise (1). Metz acknowledges that while meeting aliens, for example, would be meaningful, doing so is no more meaningful than establishing certain human connections that are in fact possible. Consider that while governments fund *some* research into exploring the possibility of extraterrestrial life, people would widely condemn governments that sought to spend most or all of their resources on such endeavours. This is because we value certain things here on earth just as much. We might make some sacrifices to search for extraterrestrial life, but many of us wouldn’t make extensive sacrifices such as giving up relationships with close friends, our spouses, or our children.^39^ Indeed, losing out on these sorts of relationships is often thought to rightly *decrease* the meaning of one’s life.

Metz’s second objection allows that in some sense the cosmic meaning as described by Benatar would be “greater than what is available to an earthly life.^40^” It, therefore, grants premise (1) and instead targets premise (3) for criticism. Recall that for Benatar, it is reasonable to regret goods that are impossible for a human on our planet to possess.^41^ However, Metz strives to provide a reason to reject the notion that we should exhibit negative attitudes towards goods we cannot possess. He does this by explicating a principle he thinks is more intuitively plausible than Benatar’s claim. His first statement of the principle is: “the more likely one would have had a good, the more reason there is for negative attitudes to its absence and the judgment that its absence is unfortunate.”^42^ He also states the principle in modal terms, and this is the formulation I will work with as the language of possible worlds will make explaining examples in this context easier. Here is the principle:

Metz’s Modal Principle: The closer the world in which one could access a benefit, the more reasonable are attitudes such as sadness, disappointment, regret when one does not acquire it.^43^

Here are five different scenarios that Metz appeals to in order to illicit intuitions in support of his modal principle:

(A) You purchased the winning lottery ticket and put it in your pocket, but then forgot about it and washed your clothes, destroying the ticket.
(B) Had you gone to the shop next door to you that you frequent to buy a lottery ticket, you would have purchased the winning ticket and claimed the prize.
(C) Had you gone to a shop an hour’s drive away that you had never visited before to buy a lottery ticket, you would have purchased the winning ticket and claimed the prize.
(D) Had you purchased a lottery ticket, it would not have won because it was for a date that had already passed and there was no prize to claim.
(E) There has never been a lottery system and there are no plans to set one up.^44^

Of course, Metz’s point is that in scenario (A) you should be extremely upset about not winning the lottery, with the level of negative attitude you feel decreasing in each scenario such that you should hardly be upset, if at all, about not winning the lottery in (E). With these intuitions in hand, Metz explains that his principle “entails that one has no reason to regret, be sad about, or be disappointed by its absence, supposing it is indeed impossible for us to attain.”^45^ This means that “while certain goods would be missing from our lives, it would not be a bad thing, at least not a cost of a sort that should lead us to avoid creating new lives.”^46^ In sum:

[T]here are different ways in which cosmic meaning could be absent, and, insofar as it is absent from our lives due to impossibility, we are unreasonable to deem its absence to be a cost that renders our lives unfortunate, regrettable, and sad and hence to provide reason to avoid creating them. To question this objection seems to require one to attest sincerely that one would have the same reactions to [A] and [E], or, more carefully, that one thinks one would be reasonable to have the same reactions toward them.^47^

## Objecting to Metz’s Criticisms of Premise (1)

In this section, I briefly argue against Metz’s rejection of premise (1) in Benatar’s argument. Metz focuses his objection on the possibility of interacting positively with aliens as a source of cosmic meaning, showing that such interactions are no more valuable than human interactions. But considering that some governments do spend vast resources on space exploration, even in the face of immense suffering and social inequality, suggests Metz is not entirely sensitive to just how much such things matter to certain people. It’s true we don’t spend all or most of our resources on space exploration but to spend the resources that we do while people on earth suffer seems to suggest such exploration means more to people than Metz realizes. NASA's annual budget is approximately 22 billion dollars per year. It’s also true that this represents only about 0.5% of the USA’s annual total spending. Yet notice that while these 22 billion dollars are spent, 828 million people were adversely impacted by hunger in 2021, with 45 million of them being extremely malnourished children.^48^ If connecting positively with other humans could confer the same type of meaning that connecting with extraterrestrials could, I submit we should not spend *any* money at all on such endeavors until every human has their basic physical needs met.

Metz says that people do not forgo important human relationships in order to pursue the discovery of extraterrestrials. While this is true of most people, it is false if meant as a universal generalization that applies to all humans. Astronauts have died on space missions, particularly during rocket takeoffs. Furthermore, many seem to make huge sacrifices with respect to their human relationships in order to explore space. Consider missions that involve spending many months at a space station. Some of the astronauts on these missions have partners, children, and close friends. While they may not completely give up such relationships, they are clearly willing to considerably sacrifice them. Here Metz might say that the fact that *most* of us do not make such sacrifices generally supports his point about what most of us find valuable. This may well be correct but consider that most of us could not qualify to be an astronaut. The standards for this profession are some of the highest of any profession in the world. Still, others who could meet the requirements will never have the opportunity to become an astronaut. My point here is only that Metz makes a claim about what humans value based on what appears to be empirical observations about how we spend resources and the sacrifices we’re willing to make, and that this observation is too quick.

Recall Metz focuses on aliens because he agrees with Benatar that God (probably) does not exist. He writes that “Benatar is open to the idea that God might in principle be a source of a cosmic meaning, but the problem for him, for me, and for many others is that the evidence of God’s existence is weak.”^49^ Given the vast amount of time, money, and energy, among other resources that humans have put into trying to connect with the divine, it’s apparent that many religious believers think that connecting with God would indeed confer cosmic meaning. Neither Metz nor Benatar deny that this is the case. But Metz seems to think that positively connecting with aliens would confer meaning similar to that had when positively connecting to other humans. I disagree as I think many believe connecting with species beyond our planet is more like connecting with the divine than with other humans. The experience of connecting with aliens whom we have never met, know nothing about, and are so wholly ‘other’ from humans, sounds a lot more like connecting with the divine than with other humans. And such a connection seems far more meaningful than the everyday ones we have with our fellow humans (or at least a significant number of people appear to think so).^50^ Metz’s objection to premise (1) fails.

Now, Metz or others might fairly respond that we are not in a good epistemic position to know what it would be like to meet an alien. Meeting an alien might turn out to be disanalogous to meeting the divine. A meeting with the divine could serve to further ground one’s values. This is not necessarily so when it comes to meeting an alien. Such a meeting could ground one’s values, but it could also serve to completely destabilize them. Perhaps from my perspective, the alien race in question has an inverted moral spectrum and believes that things like genocide, rape, and the like are morally praiseworthy. Meeting such a species could hardly be analogous to meeting the divine. Even if it didn’t detract from meaning, it would hardly add to it. Or consider an example that is less jarring. Suppose that meeting an alien turns out to be more similar to encountering an octopus or platypus for the first time. Such creatures are interesting and worth studying closely and doing so may confer meaning on one’s life. But it would surely not confer the type of cosmic meaning that is in question here (a positive impact for the universe, forever). Again, this would show that meeting the divine is not like meeting an alien. The cases are disanalogous.^51^

I Acknowledge that this objection does show that my analogy between the divine and aliens is likely too hasty. We are simply not in a good epistemic position to know what it would be like to meet an alien. In order for us to assess the type of value, there is in meeting an alien we would have to actually meet an alien. Furthermore, if there are different types of aliens, with different values associated with meeting them, we would need to meet all the types in order to know the value of such meetings. So, the problem with assigning a value to such meetings is a result of our (currently) poor epistemic position in such matters. However, this is an epistemic sword that cuts both ways. If on the one hand, I can’t be confident of the purported value of meeting with aliens, then, on the other hand, Metz cannot be confident of the disvalue of such a meeting. Since Metz and I are in equally bad epistemic positions with respect to assessing the value of meeting aliens neither he nor I should appeal to it in this discussion. Metz’s objection to premise (1) still fails.

## Objecting to Metz’s Criticisms of Premise (3)

In this section, I offer reasons for rejecting Metz’s Modal Principle. While I concede that I share Metz’s intuitions about how we should react to scenarios (A) through (E), I deny that these scenarios show that his Modal principle is true. This is because there is a powerful counterexample to his principle in the existence of death and/or significant life extension. Metz’s rejection of premise (3) fails.

### The Desirability of Immortality

Consider the inevitability of death. On metaphysical naturalism (something assumed in this argumentative context), every single person faces biological death. This is the end of one’s existence, there is no afterlife.^52^ I submit that it is right to have a negative attitude toward death. However, the possible worlds in which humans don’t die are *incredibly distant* from our own world, if they are even possible.^53^ So, according to Metz’s Modal Principle, I should not regret the fact that I will die. But since I am clearly rational to deeply regret the inevitability of my death, Metz’s Modal Principle is false.

### Boredom and Immortality

One reason to deny the fact that death is bad, and hence to deny my objection to Metz’s Modal Principle is if it turns out that living *forever* is not nearly as good as many of the major world religions claim. Bernard Williams is known for having argued that immortality cannot be necessary for a meaningful life because it would (eventually) become boring.^54^ Since a boring life cannot be meaningful, immortality turns out to not be such a great good after all. On the other hand, such boredom might also reduce the amount of meaning in a person’s life, even if it doesn’t completely negate it. In this context, then, it might be claimed that I am irrational for regretting the fact that I will die since living forever is not actually good.

Such a response to my objection, however, is not available to Metz because elsewhere he argues that Williams’s boredom objection to immortality fails.^55^ Metz denies “that boredom necessarily undercuts meaning.”^56^ He suggests that boring lives can indeed be meaningful and that one’s life doesn’t necessarily diminish in meaning as it becomes increasingly filled with boredom. One reason Metz offers for this claim is apparent when he asks us to “imagine that Mother Teresa had been bored by her work (at least in the stereotypical understanding of it). I submit that her life would have been significant, at least to some substantial degree, simply by virtue of having substantially helped so many needy people.”^57^ So, the idea here is that boredom only hinders meaning if it prevents one from taking constructive actions. The natural question, then, is to ask whether immortality would be so boring that we could no longer be constructive.^58^ But Metz also denies that being unable to take constructive actions necessarily detracts from meaning:

Suppose that [a] person had volunteered to be bored stiff so that many others would not be bored stiff. Consider, say, someone who volunteers to be head of an academic department, taking on administrative burdens, and attending dull meetings, so that his colleagues can avoid doing so. I submit (indeed, hope) that having done so would confer some meaning on his life, which means that boredom as such is not sufficient for absence of meaning.^59^

So it seems that Metz cannot consistently reject my contention that death is a counterexample to his principle by appealing to boredom.

### Repetitiveness and Immortality

Suppose for the sake of argument, however, that Metz’s objections to Williams are wrong and that immortality leads to boredom and a lack of meaning. This would show that I cannot appeal to the fact that I will not live forever as a reason to reject Metz’s Modal Principle. There is a related worry for immortality that comes in the form of *repetitiveness*.

To repeat something is to do that thing again. Might not an eternal life, with no possible limits on one’s time, eventually become repetitive in a negative way?^60^ Despite my lack of propensity for learning languages, suppose that given enough time I am able to become fluent in a language other than my mother tongue. But in a world in which I live forever, I have no time constraints. I have the time to learn more than just one additional language. Suppose I devote my time to language learning, intending to learn all of the (at least) five thousand living languages. Given plausible limits on human cognition I simply cannot retain all five thousand languages at once (nor could someone with a high propensity for languages). Imagine that I can only retain one hundred languages at a time. Once I reach one hundred, I have to jettison one language I already know in order to learn a new one. Let’s say this process happens subconsciously so I don’t have to consciously choose which language to give up. In some sense I could learn all five thousand languages, but not all at once. After enough time elapsed, I would begin relearning languages I had forgotten. I wouldn’t realize I had learnt them before since I forgot them while taking other languages on board. I would thus start to *repeat* learning languages I had known earlier. My intuition is that participating in this type of repetition is a rather undesirable state of affairs.^61^ These ideas can be applied to other forms of inquiry and also to many other types of experiences we typically think are positive. There appears to be an upper limit to these goods such that they will start becoming negatively repetitive, and this may cause one’s life to lose meaning. So, immortality is bad because while it might not be boring, it will eventually become repetitive.

One might respond that I have loaded the example by building into it the idea that I would not be able to retain all five thousand languages at once. But notice that we would have to be incredibly differently constituted creatures in order to be able to retain fluency in every single language on earth all at once. Remember that Benatar and Metz are assuming that naturalism is true. This means that the world in question is not one where we live eternally in heaven as disembodied spirits and have different abilities than we currently possess. Yet even if this were the case, there may well be an upper limit to the quantity and quality of goods that a human can possess. With enough time we might well reach that upper limit with respect to the quantity and quality of goods. While this is a worry about upper limits, not repetitiveness, it would still show that immortality is undesirable.^62^ Metz’s objection to (3) fails.

### The Desirability of Life Extension

I’m doubtful that worries about either boredom or repetitiveness are successful in showing that living forever would negate or detract from meaning in life. But let’s set this aside and suppose for the sake of argument that immortality would not be good because of boredom and/or repetitiveness. This would mean it’s hardly regrettable that I will die one day.^63^ If this is right, then Metz’s Modal Principle would not be defeated by my death counterexample. This is, however, too quick. Notice that there is a distinction between living forever and living for a significantly longer period of time. Even supposing that immortality negates or detracts from meaning, it doesn’t follow that living *significantly longer* does the same. In fact, it seems to me that the boredom or repetitiveness objections only gain traction after one has lived for many thousands, if not millions or billions, of years. In what follows I explicate why I think this is the case.

Admittedly, there may well be nearby possible worlds where we can extend human life to, say, 150 years of age. So, according to Metz, I would be justified in regretting the fact that I will not live to be that old because the worlds in which I do live that long are nearby. However, even in a world where I could live to 150 years of age, I submit that I would still be justified in regretting the fact that I cannot live much longer. Surely the possible worlds where I can live for, say, 100 thousand years are incredibly distant. The terror and sadness at the inevitability of death might not be appropriate in the face of immortality, but such reactions are appropriate in the light of not living significantly longer.

Why think that the boredom or repetitiveness objections don’t kick in much sooner than the many thousands of years I suggest? Here I appeal to work on *deep time* by J.L. Schellenberg to show that even if living forever is not something we should ultimately hope for, we are still right to want to live for many thousands (if not millions) of years.^64^ Consider that inquiry is a plausible candidate for conferring (at least some) meaning on life. Schellenberg notes that with respect to an evolutionary timescale that the human species is incredibly *young*. Homo sapiens have only been around for approximately two hundred thousand years. Yet the planet is supposed to continue for another billion years before ending in heat death (i.e., this is what scientists say would happen naturally if humans were not destroying the planet on their own). Schellenberg writes that “[e]volutionary time is of an extent almost beyond fathoming – that’s why scientists call it ‘deep’… Stephen Jay Gould, put it this way: ‘an abstract, intellectual understanding of deep time comes easily enough – I know how many zeroes to place after the 10 when I mean billions. Getting it into the gut is another matter.”^65^ Schellenberg is writing in the context of seeking to establish the claim that humans are *intellectually immature*, and hence immature in their thinking about religion. He claims that:

[O]ne needs to think hard about the fact that the perhaps 200, 000-year history of H. sapiens is wedged between three and a half billion years of evolutionary development on one side—life’s past—and another billion on the other—life’s potential future […] A billion years is a period of time ridiculously longer than the 50, 000 years of thinking and feeling that, on a generous estimate, our species has put into religion so far. What developments in religiously-relevant thought and feeling might Earth see in so much a time?... Even if we restrict ourselves to the possible future of our own species, the numbers are staggering. H. sapiens, though manifesting its religious inclinations and symbolic powers a bit earlier, has at most 6,000 years of organized and systematic religious inquiry to its credit.^66^

Notice, however, that Schellenberg’s point can be applied to all other areas of inquiry. Indeed, religion is probably one of the subjects that have been studied (in any kind of systematic way) by humanity for the longest. Thus, what Schellenberg says about religion necessarily applies to all other potential areas of inquiry.^67^ While Schellenberg is writing about our intellectual immaturity, notice what this tells us about the boredom and repetitiveness objections to immortality. In light of an evolutionary timescale, it will take an *incredibly long time* before inquiry (into any subject) becomes so boring or repetitive that it begins to detract from meaning in life. Given our intellectual immaturity, we aren’t in a good epistemic position to fathom the kind of philosophical, moral, religious, and scientific advancements that could be made one thousand years from now, let alone ten thousand, one hundred thousand, or millions of years from now. There’s much more reason to think that participating in the such inquiry will be rewarding than to believe it would become boring or repetitive anytime soon. Indeed, in my own young research career, I can already envision a meaningful research programme that would extend well beyond my natural lifespan.

In the context of the inquiry, a good is often claimed to bestow meaning on lives, boredom or repetitiveness only become a worry (if they really are one) after an incredibly long period of time. In light of this, I conclude that even if I should not regret the fact that I will not live forever, I am perfectly reasonable to regret that I will not live significantly longer than the 80 years or so that I will get (if I am lucky enough to live that long in relatively good health). The possible worlds in which I live significantly longer (i.e., many thousands or millions of years) are quite distant from our own world. This is enough to demonstrate that Metz’s Modal Principle is false. Death might not be a reason not to procreate, but the fact that our lives in the actual world are incredibly short on an evolutionary timescale is indeed great harm and something we are all quite reasonable to regret. Since Metz’s Modal Principle is false, his objections to (3) still fail. Benatar’s conclusions about cosmic meaning and anti-natalism remain unscathed.^68^

## Conclusion

According to Benatar to have cosmic meaning is to be able to transcend the space and time of our own short-lived existences on planet earth. God might provide such cosmic meaning, but Benatar denies that God exists. We also can’t interact with an alien species (even if they exist) in order to gain such meaning. The fact that we cannot have cosmic meaning is a reason in favour of anti-natalism. Instead of claiming that humans can indeed have this kind of cosmic meaning, Metz has objected that it’s actually not that significant. Positive interactions with aliens are no more valuable than those we have with humans. I responded that the fact that we spend *any* money on space exploration at all, in addition to the idea that meeting aliens may well be more like encountering the divine, supports the contrary. More importantly, since we aren’t in a good epistemic position to know the value of meeting aliens, neither Metz nor I can appeal to them in this discussion.

Metz’s Modal Principle casts doubt on the idea that we should exhibit negative emotions toward our lack of cosmic meaning since such meaning only exists in incredibly distant possible worlds. I objected to this principle on the grounds that while the worlds in which I live forever are incredibly distant from the actual world, I am right to regret the inevitable fact of my death. Even if the boredom or repetitiveness objections to immortality succeed, they still fail to show that significant life extension would not be good. Given that the human species is incredibly young in evolutionary terms, I could live many thousands if not millions of years before boredom or repetitiveness began to detract from the meaning found in inquiry. However, the worlds in which significant life extension is possible are incredibly distant from the actual world so Metz’s Modal Principle remains false. Benatar’s Argument for Anti-Natalism from Our Lack of Cosmic Meaning might be unsound, but not for the reasons suggested by Metz.^69^

## Footnotes

1 See Christopher Belshaw, “A New Argument for Anti-Natalism,” South African Journal of Philosophy, Vol. 31, No. 1 (2012): 117-127; David Benatar, Better Never to Have Been: The Harm of Coming into Existence (New York: Oxford University Press, 2006); David Benatar, “The Misanthropic Argument for Anti-Natalism,” in Sarah Hannan, Samantha Brennan, and Richard Vernon, eds, *Permissible Progeny?: The Morality of Procreation and Parenting* (Oxford: Oxford University Press, 2015): pp. 34-58; Gerald Harrison, “Antinatalism, Asymmetry, and an Ethic of Prima Facie Duties,” South African Journal of Philosophy Vol. 31, No. 1 (2012): 94-103; Asheel Singh "Furthering the Case for Anti-Natalism: Seana Shiffrin and the Limits of Permissible Harm,” South African Journal of Philosophy Vol. 31, No.1 (2012): 104-116.

2 E.g., Benatar, *Better Never to Have Been*, *op. cit;* David Benatar, *The Human Predicament: A Candid Guide to Life’s Biggest Questions* (Oxford: Oxford University Press, 2017).

3 Thaddeus Metz, “Does the Lack of Cosmic Meaning Make Our Lives Bad?” *Journal of Value Inquiry Vol. 56, No. 1 (2022): 37-50.*

4 Ibid., p. 47.

5 J.L. Schellenberg, *Evolutionary Religion* (Oxford: Oxford University Press, 2013).

6 Benatar, *Better Never to Have Been*, op. cit., pp. 30.

7 *Better Never to Have Been*, op. cit., pp. 32-35.

8 *Better Never to Have Been*, op. cit., p. 64.

9 *Better Never to Have Been*, op. cit., pp. 66-77.

10 Benatar, “The Misanthropic Argument for Anti-Natalism,” op. cit.

11 David DeGrazia, Creation Ethics: Reproduction, Genetics, and Quality of Life (Oxford: Oxford University Press, 2012); Thaddeus Metz,"Are Lives Worth Creating?” Philosophical Papers Vol. 40, No. 2 (2011): 233-255.

12 David DeGrazia, “Is it wrong to impose the harms of human life? A reply to Benatar,” Theoretical Medicine and Bioethics Vol. 31, No. 1 (2010): 317-331.

13 Ben Bradley, “Benatar and the Logic of Betterness,” Journal of ethics & social philosophy Vol. 4, No. 2 (2010): 1-6; see also Ben Bradley “Asymmetries in Benefiting, Harming and Creating,” Journal of Ethics Vol. 17, No. 1/2 (2013): 37–49.

14 Elizabeth Harman, “Critical Study of David Benatar. Better Never To Have Been: The Harm of Coming into Existence (Oxford: Oxford University Press, 2006),” Nous Vol. 43, No. 4 (2009): 776-785. See David Benatar, “Still Better Never to Have Been: A Reply to (More of) My Critics,” Journal of Ethics Vol. 17, No. 1/2 (2013): 121-151 for a response to some of these objections.

15 Christine Overall, Why Have Children? The Ethical Debate (MIT Press, 2012).

16 David Benatar, “Not ‘Not’ Better Never to Have Been: A Reply to Christine Overall,” Philosophia Vol. 47, No. 2 (2019): 353-367.

17 Kirk Lougheed, *African Communitarianism and the Misanthropic Argument for Anti-Natalism* (Switzerland: Palgrave Macmillan, 2022).

18 Harrison, “Antinatalism, Asymmetry, and an Ethic of Prima Facie Duties,” op. cit.; Seana Shiffri, “Wrongful Life, Procreative responsibility, and the Significance of Harm,” Legal Theory Vol. 5, No. 2 (1999): 117-148.; Singh, "Furthering the Case for Anti-Natalism: Seana Shiffrin and the Limits of Permissible Harm,” op. cit.

19 Harrison, “Antinatalism, Asymmetry, and an Ethic of Prima Facie Duties,” op. cit.

20 Belshaw, “A New Argument for Anti-Natalism,” op. ct. For another related argument see Hereth and Ferrucci (2021).

21 Deke Caiñas Gould, “Future Minds and New Challenge to Anti-Natalism,” Bioethics Vol. 35, No. 8 (2021): 793-800; Bartlomiej “Bartek” Chomanski, “Anti-Natalism and the Creation of Artificial Minds.” *Journal of Applied Philosophy* Vol. 38, No. 5 (2021): 870-885; Kirk Lougheed, “Future Minds are Not a Challenge to Anti-Natalism: A Reply to Gould,” *Bioethics* (forthcoming). For additional objections to anti-natalism I haven’t mentioned here, see Michael Hauskeller, “Anti-natalism, Pollyannaism, and Asymmetry: A Defence of Cheery Optimism,” Journal of Value Inquiry Vol. 56, No.1 (2022): 21-35 and Aaron Smuts, “To Be or Never to Have Been: Anti-Natalism and a Life Worth Living,” Ethical Theory and Moral Practice Vo. 17, No. 4 (2013): 711-729.

22 Thaddeus Metz, “The Meaning of Life,” The Stanford Encyclopedia of Philosophy. Edward N. Zalta, eds., (2021).

23 See John Cottingham, The Spiritual Dimension: Religion, Philosophy and Human Value (Cambridge: Cambridge University Press, 2005); William Lane Craig, “The Absurdity of Life without God,” reprinted in Joshua Seachris, eds, Exploring the Meaning of Life: An Anthology and Guide (Malden, MA: Wiley-Blackwell, 2013): 153–72.

24 See Stewart Goetz, The Purpose of Life: A Theistic Perspective (New York: Continuum, 2012) and John Cottinghamm, “Meaningfulness, Eternity, and Theism”, in Joshua Seachris and Stewart. Goetz, eds, God and Meaning (New York: Bloomsbury Academic, 2016): 123–36.

25 Metz, “The Meaning of Life,” op. cit.

26 Ibid.

27 Metz, “The Meaning of Life,” op. cit.

28 Benatar quoted in Metz, “Does the Lack of Cosmic Meaning Make Our Lives Bad?” op. cit., p. 38.

29 Metz, “Does the Lack of Cosmic Meaning Make Our Lives Bad?” op. cit., p. 38. It’s noteworthy that Metz says that he will “focus strictly on cosmic meaninglessness as a disvalue distinct from harm” (Metz, “Does the Lack of Cosmic Meaning Make Our Lives Bad?” op. cit., p. 38). He takes Benatar’s argument from our lack of cosmic meaning to anti-natalism to be distinct from welfarist considerations in support of anti-natalism. But it’s unclear why this is the case given that a lack of cosmic meaning is clearly a harm.

30 Metz, “Does the Lack of Cosmic Meaning Make Our Lives Bad?” op. cit., p. 39.

31 Ibid.

32 Ibid.

33 Ibid.

34 Ibid., p. 40.

35 Benatar quoted in Ibid., p. 39.

36 Ibid., p. 41.

37 Ibid., pp. 41-42.

38 Ibid., p. 44.

39 Ibid., p. 46.

40 Ibid., p. 47.

41 Metz notes the Rivka Weinberg has made similar claims. See Rivka Weinberg, “Ultimate Meaning: We Don’t Have It, We Can’t Get It, and We Should Be Very, Very Sad,” *Journal of Controversial Ideas* Vol. 1, No. 1 (2021): 1-22

42 Metz, “Does the Lack of Cosmic Meaning Make Our Lives Bad?” op. cit., p. 47.

43 Ibid.

44 Ibid., pp. 47-48; my lettering.

45 Ibid., p. 48.

46 Ibid.

47 Ibid., p. 49.

48 This data comes from the Food and Agriculture Organization of the United Nations. See UN Report: Global hunger numbers rose to as many as 828 million in 2021 (fao.org)

49 Metz, “Does the Lack of Cosmic Meaning Make Our Lives Bad?” op. cit., pp. 42-43.

50 One might protest here that I haven’t really offered an argument; I’ve merely asserted the sociological fact that certain groups of people highly value connecting with the divine. However, in Metz’s argument against (1), he appeals to facts about certain groups of people (i.e., countries) spend money and how much human connection appears to be valued. So, in this dialectical context I am within my rights to use the same methodology.

51 I am grateful to multiple participants at the Fourth Conference on Philosophy & Meaning in Life (2022) for raising this objection.

52 Certain forms are reincarnation may be compatible with naturalism, but Benatar clearly doesn’t have such a possibility in view. (Plus, it’s doubtful this would solve the problem I am about to pose since personal identity does not persist across a person’s different lives).

53 Though there is philosophical disagreement on whether human enhancement, including significant life extension is a good thing (eg., Nicholas Agar, Humanity's End: Why We Should Reject Radical Enhancement (Cambridge, MA: MIT University Press, 2010); Nicholas Agar, Truly Human Enhancement: A Philosophical Defense of Limits (Cambridge, MA: MIT University Press, 2013).), whether immortality is possible is at least partly an empirical question. Whether immortality is physically possible, and if it is, how close we are to achieving it is a matter of debate (e.g., de Grey, Aubrey., & Rae, Michael. (2007). Ending Aging: The Rejuvenation Breakthroughs that Could Reverse Human Aging in Our Lifetime. St. Martin's Press: London; D. John Doyle “Cryonic Life Extension: Scientific Possibility or Stupid Pipe Dream?” Ethics in Biology, Engineering and Medicine Vol. 3, No. 1-3 (2012): 9-28; Walter Glannon, “Extending the Human Life Span,” Journal of Medicine and Philosophy,” Vol. 27, No. 3 (2002): 339-354; Eric T. Juengst,. et. al., “Biogerontology, ‘Anti‐aging Medicine,’ and the Challenges of Human Enhancement,” Hastings Center Report Vol. 33, No. 4 (2003): 21-30; David G Le Couteur and Nir Barzilai, “New horizons in life extension, healthspan extension and exceptional longevity,” Age and Ageing Vol. 51, No. 8 (2022): 1-6.). To those who hold that the possible worlds where we achieve immortality are not distant, my claim by can be understood as conditional. If such worlds are distant, we would still be right to regret this fact. That this conditional can undergird the intuition driving my rejection of Metz’s modal principle shows my claims here not dependent on the empirical facts of the matter (though I do maintain naturalistic worlds where we achieve immortality are in fact quite distant).

54 See Bernard Williams, “The Makropulos case: reflections on the tedium of immortality,” in Bernard Williams, eds, *Problems of the Self* (Cambridge: Cambridge University Press, 1973): pp. 82-100.

55 Many others have also objected to Williams. See, for example: Christopher Belshaw, “Immortality, Memory and Imagination,” *Journal of Ethics* Vol. 19, No. 3/4 (2015): 323-348; Lisa Bortolotti and Yujin Nagasawa, “Immortality without Boredom,” *Ratio* Vol. 22, No. 3 (2009): 261-277; John Martin Fischer, “Why immortality is not so bad,” *International Journal of Philosophical Studies* Vol. 2, No. 2 (1994): 257-270.

56 Thaddeus Metz, *Meaning in Life: An Analytic Study* (Oxford: Oxford University Press, 2012): p. 135.

57 Ibid.

58 Ibid.

59 Ibid.

60 The idea for this repetitive objection comes from discussion with Thaddeus Metz.

61 If one suggests that the problem here is one of the limits of human cognition this seems to play right into the hands of Benatar. For the fact of our limited cognition might be a reason to refrain from procreation.

62 Furthermore, theists who have believe in a heaven might insist that God necessarily delimits the quantity and quality of goods available to humans. For there to be no such limits one might have to be God.

63 It would also not be wrong to bring someone into existence on the basis that they will face death, a feature of the human condition often cited in support of anti-natalism.

64 J.L. Schellenberg, *Evolutionary Religion*, op. cit.

65 Ibid., p. 3.

66 Ibid.

67 Even if I am wrong about this, it’s unlikely that any subject has been studied for more than, say 10,000 years.

68 Notice also that on this view while death wouldn’t constitute a reason not to procreate, that the harm of necessarily having a relatively short life could be leveraged as an independent reason in support of anti-natalism.

69 An earlier version of this paper was presented online at the *Fourth International Conference on Philosophy and Meaning of Life,* hosted by University of Pretoria in January 2022. Thanks to Thaddeus Metz for comments on an earlier draft, in addition to extensive discussion of related issues. Finally, I am also grateful to an anonymous referee for helpful feedback.

